# Supplementary material for: Psilocybin ameliorates neuropathic pain-like behaviour in mice and facilitates gabapentin-mediated analgesia
Source: Commun Biol. 2026 Apr 24;9:707. doi: 10.1038/s42003-026-10065-7 (PMC13201783; doi:10.1038/s42003-026-10065-7)
Supplement: Supplementary file 2 — Supplementary Information [file 42003_2026_10065_MOESM2_ESM.pdf]

Psilocybin ameliorates neuropathic pain-like behaviour in mice and facilitates gabapentin-mediated analgesia

Authors: Tatum Askey†[1], Daniel Allen-Ross†[1], Daniil Luzyanin[1], Reena Lasrado[2], Gary Gilmour[2], Stephen P Hunt[3], Francesco Tamagnini[1], Maqsood Ahmed[2], Gary J Stephens[1], Maria Maiarú[1]*

List of Supplementary Materials

Supplementary Figure 1. Effect of psilocybin on locomotor activity in male mice after SNI surgery.

Supplementary Figure 2. Effect of psilocybin on locomotor activity in naïve male mice.

Supplementary Figure 3. Effect of psilocybin on fecal output and body weight of male mice after peripheral nerve injury. A

Supplementary Figure 4: Effects of psilocybin and 5-HT_{2A}R blockade on static mechanical sensitivity and on spontaneous behaviours in male mice

Supplementary Figure 5: Power Calculation table

Supplementary figures

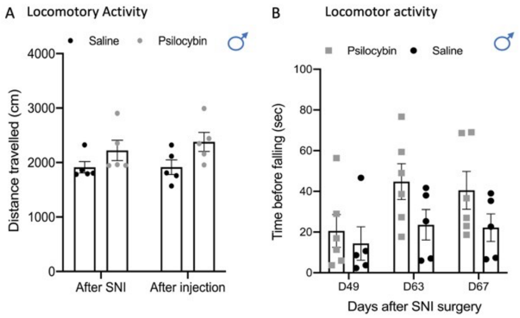

Supplementary Figure 1. Effect of psilocybin on locomotor activity in male mice after SNI surgery. A, Locomotor activity (distance travelled) after SNI surgery and subsequent injection of saline or psilocybin. Bar graph displays the total distance travelled (cm) by animals following SNI ("After SNI") and after acute injection ("After injection") of either saline (black circles) or psilocybin (grey circles). Data are presented as mean ± SEM, with individual data points shown (n=5/5). **B,** Locomotor activity measured as time before falling (in seconds) at three time points (Day 49, Day 63, and Day 67) after SNI surgery after psilocybin or saline treatment. Bars represent mean ± SEM. Individual data points are illustrated for each group at each time point.

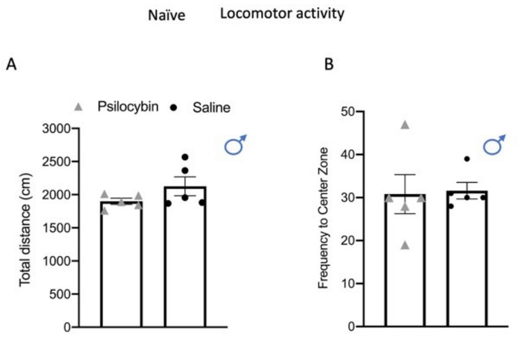

Supplementary Figure 2. Effect of psilocybin on locomotor activity in naïve male mice. The Open Field Test was used to test locomotory activity after injection of psilocybin (1 mg/kg) or saline vehicle control in naïve male mice. The total distance travelled (**A**) and the frequency to enter the center zone (**B**) were unaffected by psilocybin. n=5/5.

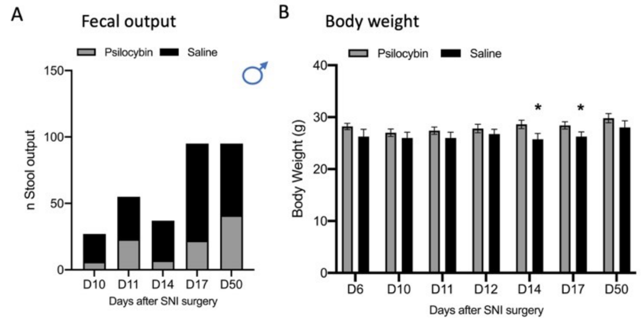

Supplementary Figure 3. Effect of psilocybin on fecal output and body weight of male mice after peripheral nerve injury. A. Total stool output was measured in male mice at different time points following spared nerve injury (SNI) surgery (days 10, 11, 14, 17, and 50). Bars represent cumulative stool counts from mice treated with psilocybin (grey) or saline (black)**. B,** Body weight was recorded before behavioural tests. SNI surgery was performed on D0, and psilocybin (1 mg/kg) or saline treatment was given on D12 after surgery. n=5/5. *P<0.05, Student's t-test. Data are expresses as mean ± SEM.

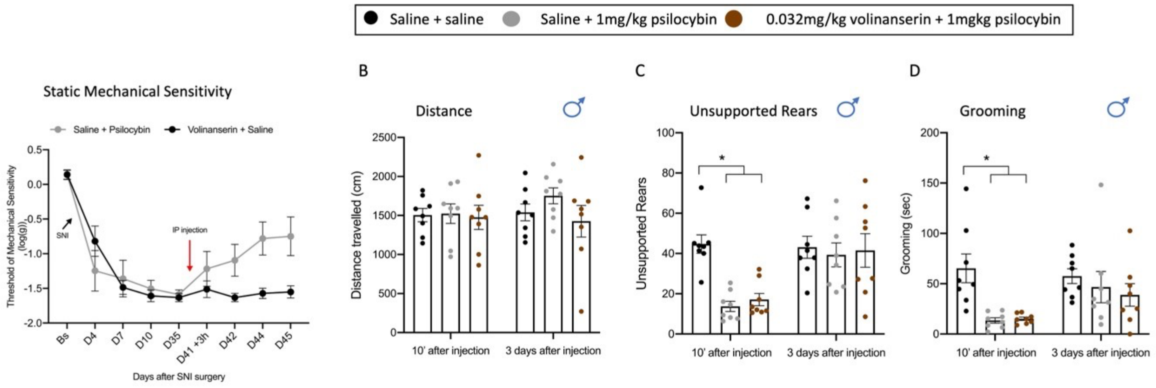

Supplementary Figure 4: Effects of psilocybin and $5\text{-}HT_{2A}R$ blockade on static mechanical sensitivity and on spontaneous behaviours in male mice

A, Static mechanical threshold of mice assessed using calibrated von Frey filaments before (BS, baseline) and after SNI surgery. On day 41 after SNI, all mice received an IP injection of saline or volinanserin (0.032mg/kg) and 30 minutes later an injection of psilocybin (0.3 mg/kg) or saline, red arrows (n=5/5). **B-D**, Male mice were injected with saline + saline (black), saline + psilocybin (1 mg/kg; grey), or volinanserin (0.032 mg/kg) + psilocybin (1 mg/kg; brown). Spontaneous behaviours were assessed either 10 minutes or 3 days after injection. (i) Total distance travelled (locomotor activity) was not significantly altered by psilocybin or volinanserin at either timepoint. (ii) Unsupported rearing was reduced 10 minutes after psilocybin administration, with or without volinanserin, but returned to baseline by 3 days post-injection. (iii) Grooming duration was similarly reduced 10 minutes after psilocybin and/or volinanserin treatment, with no persistent effects at 3 days. (n=8 pre group, two-way repeated-measures mixed-model ANOVA, factor 'treatment' 10 minutes to D3: unsupported rearing, F= 4.5, P=0.023; Tukey HSD sal+sal vs sal/psi p=0.028; sal/sal vs vol/psi p=0,07; grooming, F= 6.6, P=0.006; Tukey HSD sal+sal vs sal/psi p=0.019; sal/sal vs vol/psi p=0,01). Data are presented as mean ± SEM with individual values shown. *p < 0.05.

Figure	Two-way ANOVA	Observed Power	$\eta^2 p$
**1c male**	P=0.007	84%	0.418
**1c female**	P=0.005	88%	0.524
**1d**	P=0.034	61%	0.45
**1e**	P=0.085	41%	0.326
**1f**	P=0.002	97%	0.651
**1k**	P=0.002	91%	0.35
**2c**	P=0.004	87%	0.387
**2d**	P = 0.025	67%	0.444
**2e**	P = 0.13	32%	0.235
**2g**	P = 0.006	90%	0.631
**3b**	P=0.008	84%	0.519
**3d**	P=0.008	82%	0.407

Supplementary Figure 5: Power calculation table. Summary of two-way ANOVA results for the outcomes shown in Figures 1c–1k, 2c–2g and 3b–3d. For each panel, the table reports the omnibus two-way ANOVA P value, observed power, and partial eta-squared ($\eta^2 p$) as an index of effect size (proportion of variance explained by the effect).
