# Supplementary material for: Psilocybin ameliorates neuropathic pain-like behaviour in mice and facilitates gabapentin-mediated analgesia
Source: Commun Biol. 2026 Apr 24;9:707. doi: 10.1038/s42003-026-10065-7 (PMC13201783; doi:10.1038/s42003-026-10065-7)
Supplement: Supplementary file 3 — Description of Additional Supplementary files [file 42003_2026_10065_MOESM3_ESM.pdf]

Description of Additional Supplementary files

File name: Supplementary Data 1

Description: Numerical source data for all graphs in the manuscript can be found in supplementary data 1 file.
