# Supplementary material for: Psilocybin ameliorates neuropathic pain-like behaviour in mice and facilitates gabapentin-mediated analgesia
Source: Commun Biol. 2026 Apr 24;9:707. doi: 10.1038/s42003-026-10065-7 (PMC13201783; doi:10.1038/s42003-026-10065-7)
Supplement: Supplementary file 5 — Reporting summary [file 42003_2026_10065_MOESM5_ESM.pdf]

nature portfolio

Corresponding author(s): Maria Maiarú
Last updated by author(s): 14/10/2025

Reporting Summary

Nature Portfolio wishes to improve the reproducibility of the work that we publish. This form provides structure for consistency and transparency in reporting. For further information on Nature Portfolio policies, see our Editorial Policies and the Editorial Policy Checklist.

Statistics

For all statistical analyses, confirm that the following items are present in the figure legend, table legend, main text, or Methods section.

n/a	Confirmed	
☐	☒	The exact sample size (*n*) for each experimental group/condition, given as a discrete number and unit of measurement
☐	☒	A statement on whether measurements were taken from distinct samples or whether the same sample was measured repeatedly
☐	☒	The statistical test(s) used AND whether they are one- or two-sided<br>*Only common tests should be described solely by name; describe more complex techniques in the Methods section.*
☒	☐	A description of all covariates tested
☐	☒	A description of any assumptions or corrections, such as tests of normality and adjustment for multiple comparisons
☐	☒	A full description of the statistical parameters including central tendency (e.g. means) or other basic estimates (e.g. regression coefficient) AND variation (e.g. standard deviation) or associated estimates of uncertainty (e.g. confidence intervals)
☐	☒	For null hypothesis testing, the test statistic (e.g. *F*, *t*, *r*) with confidence intervals, effect sizes, degrees of freedom and *P* value noted<br>*Give P values as exact values whenever suitable.*
☒	☐	For Bayesian analysis, information on the choice of priors and Markov chain Monte Carlo settings
☒	☐	For hierarchical and complex designs, identification of the appropriate level for tests and full reporting of outcomes
☐	☒	Estimates of effect sizes (e.g. Cohen's *d*, Pearson's *r*), indicating how they were calculated

Our web collection on statistics for biologists contains articles on many of the points above.

Software and code

Policy information about availability of computer code

Data collection	Spontaneous behaviour was assessed using the EthoVision XT video tracking system. The EthoVision Track System was also used to record and analyze the behaviour for the open field test (OFT) . An accelerating rotarod apparatus(custum constructed) was used for analysis of ocomortory activity. No code was used in collection of data.
Data analysis	All statistical tests were performed using the IBM SPSS Statistic Programme (version 27)

For manuscripts utilizing custom algorithms or software that are central to the research but not yet described in published literature, software must be made available to editors and reviewers. We strongly encourage code deposition in a community repository (e.g. GitHub). See the Nature Portfolio guidelines for submitting code & software for further information.

Data

Policy information about availability of data

All manuscripts must include a data availability statement. This statement should provide the following information, where applicable:

- Accession codes, unique identifiers, or web links for publicly available datasets
- A description of any restrictions on data availability
- For clinical datasets or third party data, please ensure that the statement adheres to our policy

Provide your data availability statement here.

Research involving human participants, their data, or biological material

Policy information about studies with human participants or human data. See also policy information about sex, gender (identity/presentation), and sexual orientation and race, ethnicity and racism.

Reporting on sex and gender	N/A
Reporting on race, ethnicity, or other socially relevant groupings	N/A
Population characteristics	N/A
Recruitment	N/A
Ethics oversight	N/A

Note that full information on the approval of the study protocol must also be provided in the manuscript.

Field-specific reporting

Please select the one below that is the best fit for your research. If you are not sure, read the appropriate sections before making your selection.

[x] Life sciences [ ] Behavioural & social sciences [ ] Ecological, evolutionary & environmental sciences

For a reference copy of the document with all sections, see nature.com/documents/nr-reporting-summary-flat.pdf

Life sciences study design

All studies must disclose on these points even when the disclosure is negative.

Sample size	Sample size calculations were informed by variance estimates and effect sizes obtained from our previous behavioural assays, ensuring that group sizes were adequate for reliable detection of treatment effects without unnecessary animal use.
Data exclusions	No animals where excluded from the analysis
Replication	Some expriments were replicated in 2 separate litters of mice.
Randomization	Animals were assigned to psilocybin or saline randomly
Blinding	Allocation details were concealed, and cages were coded so that the experimenter performing the behavioural assessments and analyses remained fully blind to treatment conditions throughout the study. Blinding was maintained during drug preparation, testing, and statistical evaluation to minimize potential bias.<br>TA, DAR, DL and MM were blinded to treatment.

Reporting for specific materials, systems and methods

We require information from authors about some types of materials, experimental systems and methods used in many studies. Here, indicate whether each material, system or method listed is relevant to your study. If you are not sure if a list item applies to your research, read the appropriate section before selecting a response.

Materials & experimental systems

n/a	Involved in the study	
[x]	[ ]	Antibodies
[x]	[ ]	Eukaryotic cell lines
[x]	[ ]	Palaeontology and archaeology
[ ]	[x]	Animals and other organisms
[x]	[ ]	Clinical data
[x]	[ ]	Dual use research of concern
[ ]	[ ]	Plants

Methods

n/a	Involved in the study	
[x]	[ ]	ChIP-seq
[x]	[ ]	Flow cytometry
[x]	[ ]	MRI-based neuroimaging

Animals and other research organisms

Policy information about studies involving animals; ARRIVE guidelines recommended for reporting animal research, and Sex and Gender in Research

Laboratory animals	Adult male and female C57BL/6J mice (8–12 weeks old) were obtained from Charles River Laboratories (UK) and acclimatized to the housing facility at the University of Reading (UK) for at least one week prior to experimentation. Animals were group-housed in individually ventilated cages (maximum of five per cage) under standard laboratory conditions, with controlled ambient temperature and humidity. A 12:12 h light–dark cycle was maintained (lights on at 07:30 a.m.), and animals had free access to food and water throughout the study. Environmental enrichment (nesting material and shelters) was provided in all cages to promote welfare.<br>All experimental protocols were approved by the institutional Animal Welfare and Ethical Review Body (AWERB) and were carried out under UK Home Office Project Licence PPL PP9720547.
Wild animals	No wild animals were used in this study
Reporting on sex	Males and females mice were used in this study, but analysis of sex differences has not been performed.
Field-collected samples	No field collected sampled were used in this study.
Ethics oversight	Institutional Animal Welfare and Ethical Review Body (AWERB)

Note that full information on the approval of the study protocol must also be provided in the manuscript.

Plants

Seed stocks	N/A
Novel plant genotypes	N/A
Authentication	N/A
